# Atypical Femoral Fracture Following Long-Term Bisphosphonate Therapy: An Uncommon Complication of a Common Treatment

**DOI:** 10.7759/cureus.85295

**Published:** 2025-06-03

**Authors:** Diogo Ribeiro de Sene

**Affiliations:** 1 Internal Medicine, MedStar Washington Hospital Center, Washington, DC, USA

**Keywords:** atypical femoral fracture, bisphosphonate therapy, drug holiday, osteoporosis complications, subtrochanteric fracture

## Abstract

We present a case of an 82-year-old woman with an atypical femoral fracture associated with prolonged bisphosphonate therapy. After a decade of ibandronate use for osteoporosis, she sustained a subtrochanteric femoral fracture following minimal trauma. Radiographic findings were consistent with atypical femoral fracture characteristics, and surgical management with intramedullary nailing was performed. Laboratory evaluation revealed no evidence of secondary bone disease. This case highlights the importance of monitoring patients on long-term bisphosphonates and considering drug holidays to minimize the risk of atypical fractures.

## Introduction

Bisphosphonates are a cornerstone of osteoporosis management, capable of reducing the risk of typical osteoporotic fractures by roughly 50% in treated patients [1]. However, prolonged use of these antiresorptive agents has been linked to the development of atypical femoral fractures (AFFs), a rare but serious complication [1]. AFFs are unusual stress fractures that occur in the femoral shaft or subtrochanteric region with minimal or no trauma, often in patients on long-term bisphosphonate therapy [1,2]. In the general population, AFFs are very uncommon, and epidemiologic studies estimate an incidence on the order of three to nine cases per 100,000 patient-years [1]. Notably, the absolute risk of AFF rises with longer duration of bisphosphonate use, reaching on the order of ~100 per 100,000 patient-years after five to eight years of continuous therapy [3]. Despite the rarity of AFF, its occurrence has garnered significant attention because of the fracture’s severity and the need to balance this risk against the proven benefits of fracture prevention in osteoporosis [1].

While bisphosphonates are the most widely implicated medications, AFFs have also been reported with other anti-osteoporosis therapies that profoundly suppress bone remodeling. Denosumab, a receptor activator of nuclear factor kappa-B ligand (RANKL) inhibitor, appears to confer a similar risk of AFF as the bisphosphonates when used long term [4]. For example, an FDA adverse-event database analysis found significant reporting associations between AFF and both bisphosphonates (especially alendronate) and denosumab [4]. In contrast, romosozumab, a newer anabolic agent with a dual effect of increasing bone formation and reducing resorption, has shown a much lower AFF signal. Across pivotal clinical trials totaling over 5,600 patients on romosozumab, only approximately three cases of atypical femur fracture were observed [5]. It should be noted, however, that most trial patients were treatment-naïve, and there is at least one recent case report of AFF occurring after romosozumab in a patient with extensive prior antiresorptive therapy [5]. Given these considerations, clinicians in internal medicine and endocrinology should remain aware of AFF as an uncommon yet important complication of long-term antiresorptive treatment. In practice, this has prompted the adoption of “drug holiday” strategies, periodic interruptions of bisphosphonate therapy, in patients at lower fracture risk, in hopes of mitigating AFF risk while retaining skeletal benefits [1]. We present here the case of an atypical subtrochanteric femoral fracture in an older woman on long-standing bisphosphonate therapy, and we discuss the clinical features, contributory factors, and management in the context of current evidence and guidelines.

## Case presentation

**Figure 1:**
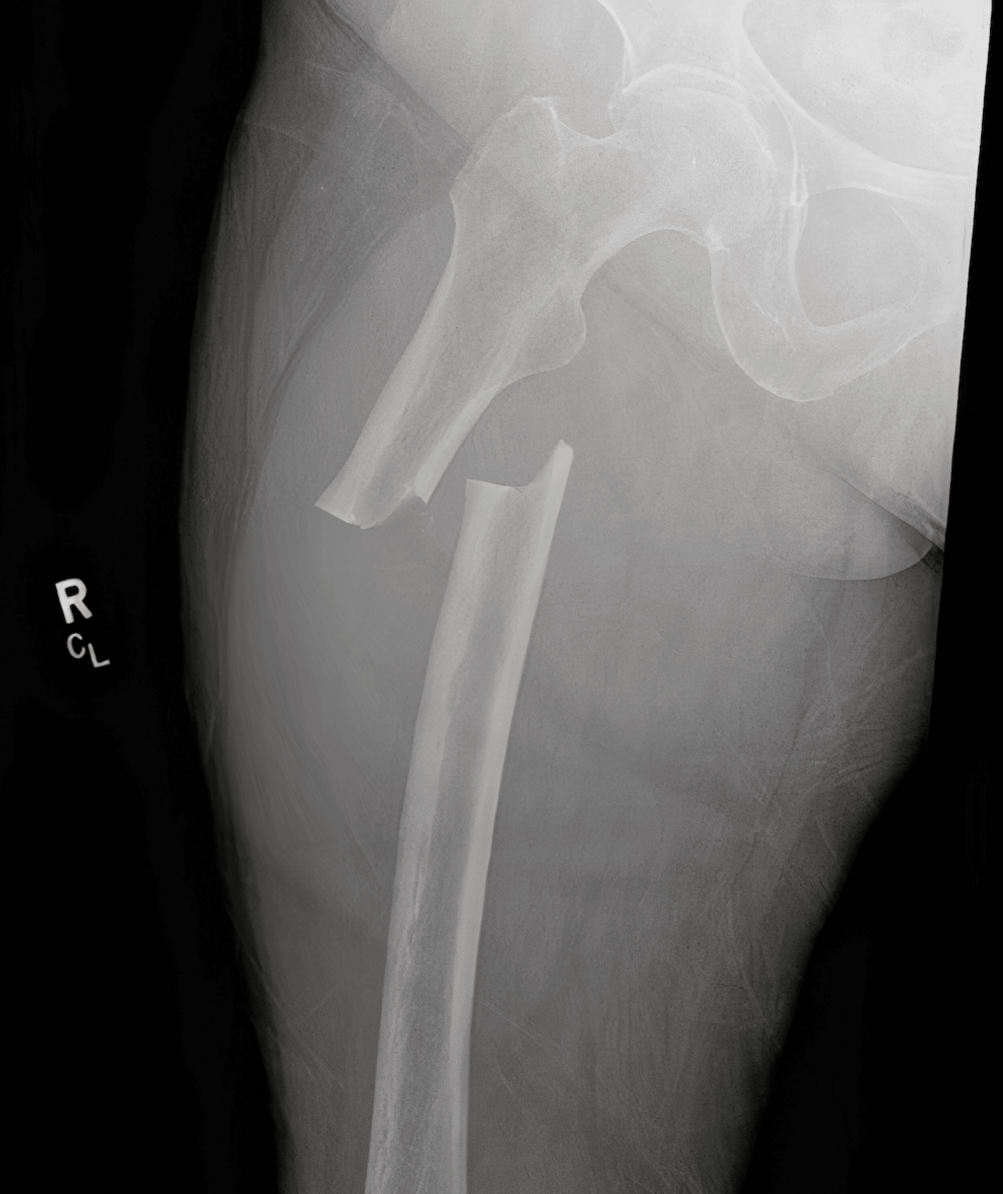
Preoperative radiograph showing a transverse subtrochanteric femoral fracture with medial cortical spike and lateral cortical thickening. R: right, CL: contralateral.

An 82-year-old woman presented to the emergency department after a low-energy fall onto her right side during a church event. She immediately noted pain in the right thigh and was unable to bear weight on the right leg. Her past medical history was significant for primary osteoporosis, diagnosed 10 years prior by bone density testing (baseline dual-energy X-ray absorptiometry showing a lumbar spine T-score of approximately -2.8). She had been treated with the bisphosphonate ibandronate continuously for 10 years. Other comorbidities included hypertension (managed with lisinopril), type 2 diabetes mellitus for 15 years (diet controlled and on metformin, with hemoglobin A1c values in the 6.5%-7% range, indicating well-controlled diabetes), and hyperlipidemia (on atorvastatin). She had no history of fragility fractures prior to this presentation. There was also no chronic glucocorticoid use or other known secondary causes of bone loss in her history. On examination, blood pressure was 135/77 mmHg, heart rate was 77 bpm, and BMI was 31 kg/m^2^; the patient was alert and hemodynamically stable. Her right upper leg was notably shortened and held in an externally rotated position, a finding consistent with a proximal femoral fracture. There was tenderness over the right thigh, but no obvious swelling or deformity of the distal leg. The neurovascular status of the right lower extremity was intact, with palpable distal pulses and intact sensation. The remainder of her physical exam was unremarkable; notably, she did not exhibit any cushingoid features such as facial fullness, dorsal fat pad, skin thinning, or easy bruising. Other signs of endocrine disorders (thyroid dysfunction stigmata, etc.) were absent. Imaging of the right femur revealed a transverse subtrochanteric fracture with a medial cortical spike and lateral cortical thickening (Figure 1), appearances that are classic for an atypical femoral fracture. Given her long history of bisphosphonate use and the radiographic features, an atypical femoral fracture (AFF) was suspected. A comprehensive laboratory workup was performed to search for any secondary contributors to bone fragility. Table 1 summarizes her key laboratory results and bone density data. Notably, serum calcium was 8.2 mg/dL (with albumin of 3.0 g/dL; the albumin-corrected calcium was within normal range), phosphate was mildly low at 1.6 mg/dL, and 25-hydroxyvitamin D was 37 ng/mL (within the optimal range of 30-50 ng/mL). Parathyroid hormone (PTH) was in the normal range, and renal function tests (blood urea nitrogen 14 mg/dL, creatinine 0.9 mg/dL) were normal. Thyroid function testing showed a normal thyroid-stimulating hormone (TSH) level (within the reference range 0.5-5.0 mIU/L), indicating that hyperthyroidism was not present. We also assessed for hypercortisolism: the patient had no clinical stigmata of Cushing’s syndrome, and a screening morning cortisol level was within normal limits, so clinically significant Cushing’s was ruled out. Importantly, her most recent dual-energy X-ray absorptiometry (DEXA) scan (performed one year before this fracture) confirmed low bone mass: the lumbar spine bone mineral density corresponded to a T-score of -2.7 and the left femoral neck T-score was -2.4, consistent with osteoporosis at the spine and osteopenia at the hip. In summary, no abnormalities suggestive of a secondary cause of osteoporosis or fracture (such as hyperparathyroidism, vitamin D deficiency, thyroid disorder, or Cushing’s syndrome) were identified in our evaluation.

**Table 1:** Laboratory results and bone density data.

Test	Result	Reference Range (Normal)		
Serum calcium (total)	8.2 mg/dL	8.5-10.5 mg/dL		
Phosphate	1.6 mg/dL	2.5-4.5 mg/dL		
Albumin	3.0 g/dL	3.5-5.0 g/dL		
Blood urea nitrogen (BUN)	14 mg/dL	7-20 mg/dL		
Creatinine	0.9 mg/dL	0.6-1.3 mg/dL		
25-hydroxyvitamin D	37 ng/mL	30-50 ng/mL (optimal)		
1,25-dihydroxyvitamin D	46.6 pg/mL	15-60 pg/mL		
Parathyroid hormone (PTH)	55 pg/mL	15-65 pg/mL		
Thyroid-stimulating hormone (TSH)	2.0 mIU/L	0.5-5.0 mIU/L		
AM cortisol (8 AM)	12 µg/dL	5-25 µg/dL		
Lumbar spine (L1-L4)	-2.7	Osteoporosis (T-score ≤ -2.5)		
Left femoral neck	-2.4	Osteopenia (T-score -1.0 to -2.4)		

**Figure 2:**
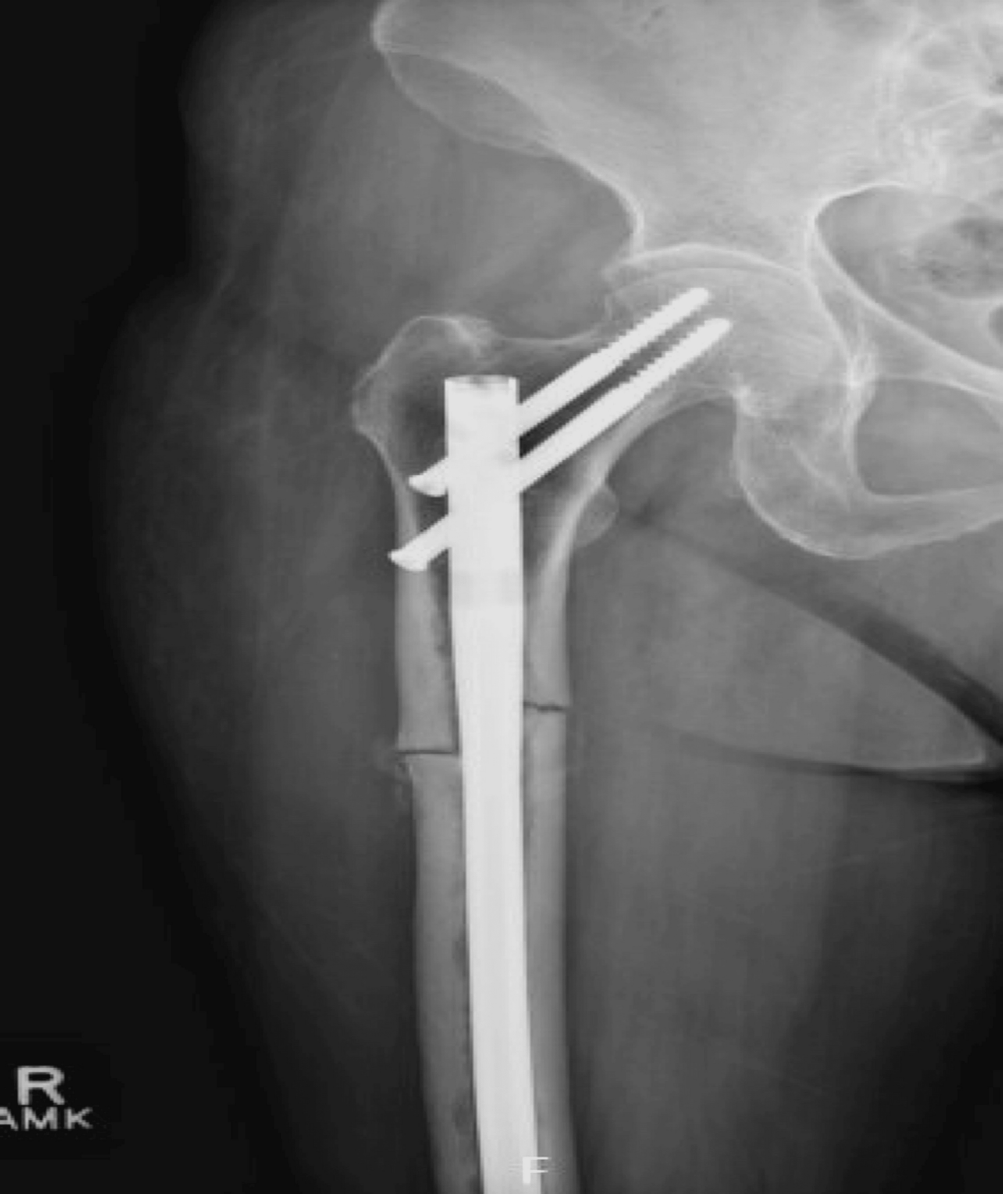
Postoperative radiograph showing intramedullary nail fixation of the subtrochanteric fracture. R: right, F: fracture, AMK: anteromedial knee.

The radiologic and laboratory findings were consistent with an atypical femoral fracture in the setting of long-term bisphosphonate use, without evidence of an underlying secondary bone disorder. The patient was managed surgically with urgent fixation of the fracture. Orthopedic surgery performed an open reduction and internal fixation using a cephalomedullary intramedullary nail (Figure 2), appropriate for a subtrochanteric femoral shaft fracture. The fracture alignment was restored, and hardware secured in good position. Her postoperative course was uncomplicated; mobilization was started with protected weight-bearing as tolerated. Ibandronate was discontinued in light of the AFF. The patient was referred to the endocrinology service for further management of her osteoporosis. The plan was to initiate anabolic therapy to improve bone density and aid fracture healing, given the contraindication to continued bisphosphonate therapy. Teriparatide (recombinant PTH 1-34) was considered as a potential next-line treatment. She was also counseled on adequate calcium and vitamin D intake and instructed to follow up for repeat bone density assessment after completion of fracture healing.

No abnormalities suggestive of secondary bone disease were identified. The patient underwent surgical fixation with a trochanteric cephalomedullary intramedullary nail (Figure 2). Bisphosphonate therapy was discontinued, and she was referred to endocrinology to initiate anabolic osteoporosis therapy.

## Discussion

This case illustrates an atypical femoral fracture (AFF) occurring after long-term bisphosphonate therapy, underscoring the need to remain vigilant for this rare complication. Atypical femoral fractures are defined by a characteristic pattern: they usually involve the femoral shaft (subtrochanteric or diaphyseal region) and have a transverse or short oblique configuration without comminution, often accompanied by cortical thickening at the fracture site [1]. Clinically, AFFs often occur with minimal or no trauma and may be preceded by prodromal thigh pain in many cases [1]. In our patient, the subtrochanteric location, clean transverse break with a medial spike, and lateral cortical thickening on X-ray were hallmark features that pointed toward an AFF rather than a typical osteoporotic femur fracture. Her history of a decade of bisphosphonate use was a major risk factor. It is worth noting that her biochemical work-up (normal calcium, phosphate, PTH, vitamin D, renal and thyroid function) effectively ruled out common secondary causes of bone fragility, such as primary hyperparathyroidism, osteomalacia, renal osteodystrophy, or thyrotoxicosis. The absence of any clinical or biochemical evidence of Cushing’s syndrome also helped exclude hypercortisolism as a contributor. Thus, the AFF was most plausibly related to long-term antiresorptive therapy in the setting of primary osteoporosis.

Pathophysiologically, long-term bisphosphonate therapy is thought to contribute to AFF through oversuppression of bone remodeling. Bisphosphonates potently inhibit osteoclast-mediated bone resorption, which over time can lead to the accumulation of microdamage in bone because new bone formation (coupled to resorption) is also reduced [2]. Normally, microscopic cracks in bone are repaired by targeted remodeling, a process by which osteoclasts resorb the damaged areas, allowing osteoblasts to lay down new bone [2]. In the presence of potent antiresorptives, this repair mechanism is blunted, and microcracks may coalesce and propagate, eventually manifesting as stress fractures [2]. Biomechanical studies have also suggested that bisphosphonates can alter bone material properties: cortical bone from patients on long-term bisphosphonates shows increased mineralization homogeneity and reduced toughness, which could make bone more brittle despite preserved density [1]. These factors together provide a plausible explanation for why a subset of patients on prolonged therapy experience atypical fractures in areas like the femoral shaft, where tensile stresses from daily activities (e.g., walking) can facilitate crack growth once a defect has formed.

Aside from medication exposure, patient-specific factors also influence AFF risk. Epidemiologic data have identified a predisposition in certain populations; for example, Asian ethnicity has been associated with a significantly higher incidence of AFF among bisphosphonate users, on the order of four- to eight-fold greater risk than in white populations [1]. The etiology of this racial disparity is not fully understood; differences in hip geometry have been postulated as one contributing factor. Patients who sustain AFF often have distinct femoral geometry, such as a greater lateral femoral bowing or a more acute femoral neck-shaft angle, which increases lateral cortical stress [1]. Our patient did not have any obvious anatomic abnormalities noted, but advanced age (over 80) and female sex likely added to her risk burden, as most AFFs occur in older women [3]. Concurrent medications and comorbidities can also play a role. Glucocorticoid use is a well-known risk factor for osteoporosis and has independently been linked to atypical fractures [1]. Proton pump inhibitors, which can impair calcium absorption, have also been associated with AFF in some studies [1]. Our patient was not on these medications, but this is a relevant consideration in general. Conditions such as rheumatoid arthritis and diabetes mellitus have been reported in higher frequency among AFF patients as well [1], possibly reflecting an overall higher fracture risk profile or effects of those diseases on bone quality. In summary, AFF is usually a multifactorial event; prolonged antiresorptive therapy is the key driver, but anatomical factors, genetic predispositions, and additional drugs or diseases may modulate the risk in any given patient.

With growing awareness of AFF over the past decade, there has been an emphasis on preventive strategies and judicious use of long-term bisphosphonates. Current clinical guidelines endorse the concept of a bisphosphonate “drug holiday” for certain patients. In those with low-to-moderate fracture risk, it is recommended to consider an interruption of therapy after approximately three to five years of continuous bisphosphonate use [1]. This approach is supported by observations that AFF risk increases markedly beyond five years of use [1] and that the risk appears to decline rapidly after stopping therapy [3]. For example, Schilcher et al. noted that the relative risk of AFF drops by about 70% for each year off bisphosphonates after discontinuation [3]. Therefore, in a patient who has been treated for several years and now has improved bone density or no new fractures, a temporary halt in therapy may be prudent to allow bone remodeling activity to recover. During a bisphosphonate holiday (often two to three years in duration), patients should be periodically re-assessed with clinical evaluation, bone turnover markers if appropriate, and DEXA scans to ensure skeletal stability [1]. On the other hand, patients at high fracture risk (for instance, those with very low bone mineral density (BMD) (T-score ≤ -2.5 at the hip or spine), prior history of osteoporotic fractures, advanced age, or other strong risk factors) are often advised to continue therapy longer, as the benefit of preventing common fractures in these individuals usually outweighs the small absolute risk of AFF [1]. In our patient’s case, she was maintained on bisphosphonates for 10 years due to severe osteoporosis and advanced age, putting her in a higher risk category for future fractures; unfortunately, this extended treatment duration also increased the probability of AFF. This case thus highlights the importance of ongoing re-evaluation: after about five years of therapy, clinicians should weigh the patient’s current fracture risk against the accumulating AFF risk and consider modifying the treatment plan accordingly [1]. Management of an AFF, once it occurs, requires both surgical and medical approaches. Surgical fixation is indicated for complete fractures (like the transverse break in our patient’s femur) because these fractures have poor inherent stability and a high risk of complications if treated conservatively. Intramedullary nailing is the preferred surgical technique, as it provides durable internal support along the length of the femur. Our patient underwent intramedullary nail fixation, which is standard and has a high likelihood of restoring function while the fracture unites. Even in the case of incomplete AFFs (stress reactions or cracks that have not progressed to full fractures), prophylactic surgical fixation is often recommended if the lesion is accompanied by pain or is substantially through the cortex, since progression to a complete fracture is common and can be catastrophic. In our patient, the fracture was complete, so prompt surgical management was appropriate. From a medical management standpoint, the priority is to halt the offending antiresorptive therapy and promote fracture healing. All antiresorptive agents (bisphosphonates, denosumab) should be discontinued in the setting of an AFF. Ensuring adequate calcium and vitamin D nutrition is critical for skeletal healing, and supplements should be provided if needed. There is also increasing interest in using anabolic osteoporosis therapies to aid recovery and improve bone strength after AFF. Teriparatide (recombinant parathyroid hormone 1-34) is an anabolic agent that stimulates osteoblast activity and increases bone turnover, essentially the opposite action of bisphosphonates. Several case series and studies suggest that teriparatide can accelerate the healing of atypical femur fractures. A 2020 systematic review by the European Calcified Tissue Society found that among patients with AFF treated with teriparatide, a substantial proportion achieved radiographic fracture union within six months, especially in those who had surgical fixation [6]. In that review, approximately 75% of complete AFFs united within six months of teriparatide therapy, and healing rates were also favorable for incomplete fractures (over 40% healed in six months with teriparatide alone in cases managed conservatively) [6]. These observational outcomes suggest a potential role for teriparatide in enhancing AFF healing. Our patient was accordingly referred for consideration of teriparatide after surgery. It must be noted, however, that high-quality evidence is limited. There have been no randomized controlled trials in this scenario, and thus, the strength of recommendation for teriparatide is based on case reports and expert opinion rather than definitive proof [6]. The 2020 expert panel concluded that there is no absolute, evidence-based mandate to use teriparatide specifically for AFF, apart from the general benefit it provides in improving bone density and reducing standard osteoporotic fractures [6]. Nonetheless, they acknowledged that teriparatide “might” result in faster healing of surgically treated AFFs and incorporated it as a consideration in post-AFF management algorithms [6]. In practice, many clinicians will initiate an anabolic drug (such as teriparatide or the newer PTH analog abaloparatide) after an AFF, particularly if the patient remains at high risk for fragility fractures but cannot resume bisphosphonates or denosumab. Anabolic therapy can both aid fracture healing and help rebuild bone mass that may have been lost during any drug holiday period [6].

Other medical therapy options may be tailored to the individual’s risk profile. For patients who have experienced an AFF but still have a need for osteoporosis treatment, selective estrogen receptor modulators (SERMs) like raloxifene may be considered in certain cases, for example, in younger postmenopausal women at lower risk of hip fracture [6]. Raloxifene has not been associated with AFF and provides modest antiresorptive effects mainly for vertebral fracture reduction. Hormone replacement therapy or calcitonin could be alternative considerations in specific scenarios where first-line osteoporosis drugs are contraindicated [6]. On the other hand, continuing a potent antiresorptive such as denosumab in an AFF patient is generally avoided unless no other options exist, because there have been reports of recurrent or contralateral AFFs in patients who were switched from bisphosphonates to denosumab after an initial AFF [6]. In our patient’s care, the plan was to use teriparatide to rebuild bone strength, with the hope that after a year or two of anabolic therapy, we could then reassess the need for any further anti-osteoporosis treatment. If she remains very high risk (for example, if her bone density remains very low or she has new fractures), transitioning to an agent like romosozumab for a limited course could be contemplated, as romosozumab provides anabolic effects with a shorter duration of antiresorptive activity and has not demonstrated a strong AFF signal in clinical trials [5]. Ultimately, management after AFF requires a personalized approach that weighs the risks of future typical fractures against the risk of another atypical fracture.

In conclusion, this case reinforces several important points for clinicians. First, atypical femoral fractures, though uncommon, should be on the differential when an osteoporosis patient presents with thigh or groin pain, or with a femoral shaft fracture after little or no trauma. Early recognition of AFF is crucial: patients often report prodromal pain, and if an incomplete fracture is caught in time (e.g., via X-ray or MRI for thigh pain), surgical prophylaxis can prevent a complete break. Second, the duration of antiresorptive therapy is a key risk factor; continual bisphosphonate use beyond five years should prompt periodic re-evaluation of the ongoing need for therapy. For many patients, a planned drug holiday can be instituted to reduce AFF risk, as current guidelines recommend in low-risk scenarios [1]. Third, if an AFF does occur, management entails cessation of bisphosphonates, surgical repair if indicated, and a shift in osteoporosis treatment strategy. Anabolic agents like teriparatide have emerged as a valuable tool in this context, offering a chance to enhance fracture healing and restore bone strength, although definitive evidence is still forthcoming [6]. Lastly, continued research and vigilance are needed: as new osteoporosis medications (such as romosozumab) become available, we must monitor for long-term effects on bone quality. The balance between treating osteoporosis to prevent common fractures and avoiding rare complications like AFF is delicate. Through individualized care, selecting appropriate candidates for prolonged therapy vs. drug holidays, and ensuring close follow-up, clinicians can optimize bone health outcomes while minimizing the risk of atypical fractures in their patients.

## Conclusions

Atypical femoral fractures are a rare but increasingly recognized complication of prolonged bisphosphonate therapy. This case underscores the importance of maintaining clinical vigilance in patients presenting with unexplained thigh pain or low-energy femoral fractures, especially those on long-term antiresorptive treatment. Prompt identification, cessation of bisphosphonates, and appropriate surgical management are essential for optimal outcomes. Furthermore, this case reinforces the need for individualized treatment duration and periodic reassessment of fracture risk, including consideration of drug holidays, as part of a comprehensive osteoporosis management strategy. Increased awareness and early intervention can help mitigate morbidity and improve long-term skeletal health in patients receiving bisphosphonates.
